# Frequency and Correlates of Fathers' Accommodation in Pediatric Anxiety Disorders

**DOI:** 10.1007/s10578-021-01190-x

**Published:** 2021-05-19

**Authors:** Ena Alcan, Tess Anderson, Eli R. Lebowitz

**Affiliations:** 1 Department of Clinical Psychology, Experimental Psychopathology, and Psychotherapy, Institute of Psychology, Philipps-University Marburg, Gutenbergstraße 18, 35037 Marburg, Germany; 2 Yale Child Study Center, Yale School of Medicine, New Haven, CT USA

**Keywords:** Anxiety disorders, Family, Accommodation, Fathers

## Abstract

Previous studies investigating family accommodation (FA) in pediatric anxiety disorders have primarily relied on mothers' reports, while data on FA by fathers remains scarce. We examined the frequency and correlates of fathers' FA of anxious children and compared fathers’ and mothers’ reports of FA. Participants were 69 parents of treatment-seeking children and adolescents with a primary anxiety disorder. FA was highly prevalent amongst fathers, with the majority of fathers participating in symptom-related behaviors and modifying family routines due to child anxiety. Fathers' accommodation levels were significantly correlated with fathers' reports of child internalizing symptoms, child externalizing symptoms, and fathers' own anxiety symptoms. Fathers’ and mothers’ reports of FA were moderately correlated, whereas their reports of their respective distress related to the need to accommodate were only weakly correlated. Fathers reported a significantly lower frequency of FA than did mothers. These findings highlight the importance of obtaining reports from both fathers and mothers when assessing FA. Results are particularly relevant to family-focused and parent-based interventions designed to address and reduce FA amongst parents of clinically anxious children.

## Introduction

Anxiety disorders are among the most common disorders in childhood and adolescence [1, 2], with lifetime prevalence estimates as high as 28.8% and 31.9% [3, 4]. Anxiety disorders usually onset in childhood, with a median age of onset of 6 years [4]. If not treated, anxiety disorders often persist into adulthood [5], causing significant short- and long-term impairment in social, academic, and occupational functioning [1, 6–8], with substantial societal cost [9–11]. In pediatric anxiety research, considerable attention has been focused on investigating the role parental behaviors play in the development, maintenance, and treatment of pediatric anxiety disorders [12–17], with a substantial number of studies focusing on a particular cluster of parental behaviors known as family accommodation (FA) [18, 19].

FA describes the ways in which family members of children with psychiatric disorders change their own behaviors in response to the symptoms exhibited by the child [19, 20]. In pediatric anxiety disorders, FA refers to both active participation by parents in symptom-driven behaviors and to modifications that parents make to family routines, scheduled work and travel plans, and leisure activities to help a child alleviate or avoid distress caused by anxiety [19, 21]. The desire to reduce child distress caused by anxiety symptoms has been identified as a major motivating factor driving FA [22]. As parents attempt to mitigate or avoid a child's distress, they facilitate the child's avoidance of anxiety-provoking situations, thereby reinforcing the reliance on parents, maintaining the anxiety symptoms, and hampering the development of more independent coping strategies [19, 23]. Some typical examples of FA in pediatric anxiety disorders include: providing repeated reassurance in generalized anxiety disorder, sleeping next to the child in separation anxiety disorder, speaking in place of the child in social anxiety disorder, and facilitating avoidance of feared objects in specific phobias [19, 21, 24].

Several previous studies have suggested that greater FA is associated with worse symptom severity [19], more functional impairment [21, 25, 26], and poorer treatment outcomes [27–30]. For example, in a study of treatment response in children and adolescents with obsessive–compulsive disorder (OCD), FA emerged as a significant predictor of poorer response to CBT and pharmacological treatment of pediatric OCD [27]. Similarly, several studies have shown that addressing or reducing FA during treatment significantly improved treatment outcomes in children and adolescents with anxiety disorders and OCD, thereby identifying FA as an important treatment target that could potentially improve existing interventions and enhance treatment outcomes and gains over time [18, 28–31]. In fact, a meta-analysis of 29 studies found that family treatments that addressed FA in OCD significantly improved the functioning of OCD patients compared to family treatments that did not address FA [32]. However, despite the substantial evidence showing that FA plays an important role in OCD and pediatric anxiety disorders, fathers have been relatively less included in FA research. In studies that have included fathers, the sample size was too small for separate effects to be tested. For example, in Benito et al. [33] 5.7% of participants were fathers, in Kagan et al. [30] only 3%, in Meyer et al. [34] 5.4% to 12.4% (depending on site), and Storch et al. [25] reported on parents combined and did not distinguish between fathers' and mothers' reports. Such underrepresentation of fathers in FA research significantly impedes our understanding of the effect fathers’ accommodation may have on the maintenance of children’s anxiety disorders and the effect on the treatment interventions designed to reduce FA of symptoms.

Although previous studies investigating FA in pediatric anxiety disorders have shown it to be highly prevalent, the majority of these studies have focused on mothers, with 95–100% of mothers reporting frequent (e.g., 3–6 times a week or daily) engagement in some form of FA [19, 25, 31]. A study by Lebowitz et al. (2014) compared the frequency of FA in clinical and non-clinical samples and found FA was more prevalent in the clinical sample, with mothers of children and adolescents with anxiety disorders and OCD reporting accommodation significantly more than mothers of non-anxious children [35]. In contrast to studies on mothers, studies reporting on fathers’ prevalence of FA are lacking. So far, only one study compared the prevalence of fathers’ and mothers’ accommodation [21]. That study assessed 68 mothers and 51 fathers using the Family Accommodation Checklist and Interference Scale (FACLIS), which was designed to assess the forms of accommodation parents provide to their anxious child, as well as the scope and interference caused by the accommodation [21]. The study found that 97% of mothers and 88% of fathers reported at least one form of accommodation, whereas no significant differences were found between mothers' and fathers' accommodation scope scores, as well as total and mean accommodation interference scores.

Studies of the clinical correlates of family accommodation have also focused on mothers [19, 31, 35], and less is known about clinical correlates of fathers’ accommodation. For example, existing studies assessing maternal accommodation have identified internalizing, externalizing, and depressive symptoms in children as significant child-related correlates [25, 31, 36, 37]. These studies found that both clinically anxious children and children with OCD engage in externalizing behaviors, such as rage attacks, outbursts, and temper tantrums, to maintain parental accommodation [18, 25, 37–39]. Indeed, mothers frequently report negative short-term consequences when refusing to accommodate their child's anxiety symptoms, such as increased anger and distress, while children report that accommodation makes them feel less anxious and wish their mothers to continue accommodating [19, 23, 25, 40]. In contrast, only one recent study on correlates of FA included a subset of fathers (*N* = 41), showing that both fathers’ and mothers’ perceptions of child emotion and distress factors (e.g., internalizing and externalizing symptoms) predicted parental accommodation over and above fathers’ and mothers’ own emotion and distress factors [41].

Similarly, studies of child-related demographic correlates of FA have focused predominantly on mothers. In a review of 69 articles on FA in child anxiety disorders, mixed findings have been reported on child sex and age and mothers’ FA [22]. Despite some data showing mothers of female children accommodating more [19], this finding has not been replicated [21, 25, 36]. Similarly, while some studies reported a significant negative association between mothers’ FA and child age [21, 25, 36], this finding has also not been consistent, with other studies reporting no relations between mothers’ FA and child age [19, 30, 34, 42]. However, in a study of FA in young children (aged 4–7) with anxiety disorders, 100% of mothers reported frequent accommodation, and a significant positive association emerged between FA and children's externalizing behaviors [31]. The authors suggested that mothers of young children may be more protective and prone to accommodate to prevent both the child's distress and their externalizing behaviors, such as outbursts and temper tantrums. To date, only one study examined the relationship between fathers’ accommodation and child sex and age, finding a non-significant association between fathers’ FA and child sex, as well as fathers’ FA and child age [41]. More research is needed in order to understand if fathers, just as mothers, are more prone to accommodate younger children.

Investigation of the parent-related correlates of FA has been similarly concentrated on mothers' accommodation [19, 31, 33, 40], whereas data on father-related correlates of FA is scarce [25, 30, 34]. In mothers, greater frequency of accommodation was positively associated with maternal distress related to accommodation [40], as well as with maternal distress in general, including self-reported maternal anxiety and depressive symptoms [21, 33, 34]. Only one study investigating fathers found no significant relationship between fathers’ FA and fathers’ self-reported anxiety and depressive symptoms [41]. Mothers' accommodation has also been linked to the severity of child distress in anxiety-provoking situations [42] and to maternal beliefs about anxiety and accommodation. For example, mothers who held negative beliefs regarding their child's experience of anxiety symptoms were more likely to respond to child distress by accommodating, as were mothers who believed that accommodation prevents a child from losing behavioral and emotional control [34, 42]. No studies thus far examined fathers’ beliefs regarding a child’s experience of anxiety or accommodation. Furthermore, parental emotional regulation was also investigated as a correlate of FA. For example, while a study by Kerns et al. (2017) found that mothers' difficulties in emotion regulation during their child's anxiety-related distress predicted the level of accommodation [43], O’Connor et al. [41] found no association between fathers’ emotional regulation difficulties and fathers’ FA. Parental anxiety and emotion dysregulation may play an important role in the accommodation of child anxiety, as children may learn from their parents and develop less adaptive regulation strategies, leading to avoidance of anxiety-provoking situations and continued dependence on the parents for accommodation [44].

The underrepresentation of fathers in the FA literature is not surprising, as the focus on mothers is typical of an overall trend in child psychiatry research. For example, a review of 577 articles in clinical child research found that fathers were significantly underrepresented compared to mothers, with only 1% of studies including only fathers compared to 48% of studies including only mothers [45]. In another review, Phares et al. [46] revealed a similar trend in pediatric and developmental psychopathology research, with fathers being vastly underrepresented. Similarly, there has been a lack of inclusion of fathers in cognitive-behavioral therapy and family-based interventions, with fathers being included in only 6.3% of therapy sessions with children [47, 48]. When considering parent-based intervention targeting FA, a most recent study showed that fathers attended only 12% of therapy sessions, whereas mothers were required to attend all the sessions [49]. Such underrepresentation of fathers in child psychiatry research is a notable limitation, as fathers’ parenting behaviors have been found to be as important as mothers’ in the development and maintenance of pediatric anxiety disorders [50–56], and in a recent study, the association between parenting behaviors and a child’s anxiety symptoms was found to be stronger for fathers than for mothers [57]. Similarly, the underrepresentation of fathers in FA research and parent-based interventions is a significant limitation considering the detrimental impact FA has on childhood anxiety disorders, including worse anxiety symptom severity, increased functional impairment, and poorer treatment outcomes. Considering that greater frequency of mothers’ FA has been found to be a significant burden on mothers, the research on FA would greatly benefit by additionally investigating underlying mechanisms involved in fathers’ FA.

Overall, not distinguishing between mothers and fathers and predominant reliance on mothers as informants in previous studies on FA is a significant limitation because of an underlying assumption that both parents share the same perspective on their child’s anxiety.

Previous studies that have investigated differences between fathers’ and mothers’ reports on child anxiety found that fathers provided a different perspective on their child’s anxiety [58–61], which is why including fathers as informants in FA research may significantly enhance the accuracy of the FA assessment that could, in turn, improve existing interventions and treatment outcomes. The present study sought to address these limitations and to advance understanding about fathers’ FA, including possible differences between fathers’ FA and mothers’ FA. Therefore, this study’s first objective was to examine the prevalence and frequency of fathers' accommodation, including participation in symptom-related behaviors, modifications of schedules and routines, fathers' distress related to the accommodation, and negative short-term child consequences of fathers not accommodating. We expected to find high accommodation rates among fathers, similar to the highly prevalent accommodation rates consistently reported by mothers [19, 21, 40].

The second objective was to compare fathers' and mothers' reports of FA. Specifically, the aim was to examine the degree to which fathers’ reports of their own accommodation are correlated to mothers’ reports of their own accommodation. Based on previous research showing a poor-to-moderate correlation between fathers' and mothers' reports of child anxiety symptoms [23, 62–64] and the moderate association between fathers' and mothers' reports of accommodation in Thompson-Hollands' study [21], we hypothesized that fathers’ accommodation and mothers’ accommodation would be correlated to a moderate degree. In addition, our aim was to examine whether there are significant differences in how much fathers accommodate compared with mothers. As previous findings show that fathers report less child anxiety than mothers, we expected that fathers would also report less accommodation than mothers.

The third objective was to examine the relations between fathers' and mothers’ accommodation and several demographic and clinical characteristics, including child internalizing and externalizing symptoms and the fathers’ and mothers’ own anxiety symptoms. Given the inconsistent findings reported on the association between FA and child age [19, 25, 30, 41, 42], no hypotheses were made about fathers’ FA or mothers’ FA. Based on several studies that did not find a link between FA and child sex [21, 25, 36, 41], we did not expect fathers' and mothers’ accommodation to differ for female and male children. Similarly, as Lebowitz et al. [19] did not find a significant association between socioeconomic status and FA, we expected that socioeconomic status would not be associated with fathers' and mothers’ reports of accommodation. Based on findings revealing significant, positive correlations between FA and child internalizing and externalizing symptoms [22, 25, 31, 41] in mothers, we hypothesized that fathers' and mothers’ reports of FA would both be positively associated with their reports of their child’s internalizing and externalizing symptoms. Finally, in light of several previous studies implicating maternal anxiety in mothers' reports of child anxiety and FA [21, 33, 34, 40, 62], we hypothesized that mothers’ reports of FA would be positively correlated with mothers’ self-reported anxiety symptoms. We expected similar findings for fathers: a positive association between fathers’ self-reported anxiety symptoms and fathers’ FA of their child’s anxiety symptoms.

## Methods

### Participants

Participants were 69 parents of treatment-seeking children (age range 6–16, $$M=9.29, SD=2.68)$$ who presented at a university-based, specialty anxiety research clinic in the eastern United States. For the purposes of this study, both fathers and mothers of treatment-seeking children had to participate in order to be included in the study.

Participating children were referred by mental health and primary care providers, school personnel, or were self-referred. Children met DSM-5 criteria for the following primary anxiety disorders: generalized anxiety disorder $(\mathrm{36,2} \%),$ social phobia $(27.5\%)$, separation anxiety disorder $(17.4\%)$, specific phobia $(10.1\%)$, agoraphobia $(1.4\%)$, and selective mutism $(1.4\%)$. Non-anxiety co-primary diagnoses (i.e., rated as interfering as the primary anxiety diagnosis) included oppositional defiant disorder $(1.4\%)$ and obsessive–compulsive disorder $(1.4\%)$. Other comorbidities including anxiety and other diagnoses were also common in this sample (one diagnosis, *N* = 54, two diagnoses, *N* = 40, three diagnoses, *N* = 19, four diagnoses, *N* = 9, five diagnoses, *N* = 3, six diagnoses, *N* = 1). Such comorbidity is common in clinical samples [25, 31, 36]. Diagnoses were established using the Anxiety Disorders Interview Schedule for Children—Parent and Child Versions, a semi-structured diagnostic interview conducted by clinical psychologists or graduate-level trainees [65, 66].

Sixty percent of children were male $\left(60.9\%, n = 42\right)$ and thirty-nine percent were female $(39.1\%, n = 27)$. The majority of children were identified by their parents as White ($84.1\%, n = 58)$ and non-Hispanic $(84.1\%, n = 58).$ A minority ($14.5\%$) of children were identified as Hispanic $(n = 10),$ and $11.6\%$ of children were identified as multiracial $\left(n = 8\right),$ and $2.9\%$ were idetified as Asian $(n = 2).$ The majority of children $(94.2\%, n = 65)$ were born in the United States, and English was the primary language spoken at home for most participants.

The majority of participating fathers $$(65.2\%)$$ and mothers ($$80.3\%$$) identified as non-Hispanic. Most fathers ($$87.3\%$$) and mothers ($$81.7\%$$) identified as White. Similarly, the majority of parents were highly educated and employed, with $$73.2\%$$ of fathers and 71.8% of mothers having a bachelor's degree or higher and $$80.3\%$$ of mothers and 95.8% of fathers being employed. Forty percent of families reported an annual family income of over $150,000 ($$40.6\%$$). The remainder of families reported household income over $100,000 annually ($$26.1\%$$) or below $100,000 ($$26\%$$).

### Procedure

The Institutional Review Board (IRB) approved the study, and parents and children provided signed informed consent and assent, respectively, before other study procedures. Trained research assistants administered all questionnaires, and clinical psychologists or graduate-level trainees conducted the diagnostic interviews.

### Measures

#### Demographic Questionnaire

Parents completed a brief questionnaire providing standard demographic information, including parents' race, ethnicity, education level, employment status, and annual household income. Similarly, parents provided information on their child's sex, age, race, and ethnicity.

#### ***Anxiety Disorders Interview Schedule—Child and Parent Version (ADIS-C/P)***[66]

The ADIS-C/P is a semi-structured interview used for establishing the presence and severity of primary anxiety disorders and related psychopathology in children and adolescents. The ADIS-C/P is the most widely used instrument for diagnosing anxiety disorders in children and adolescents. The diagnoses are established according to DSM-5 diagnostic criteria, with the severity of impairment rated on a 9-point scale, from 0 to 8, and a rating of 4 or higher, indicating the presence of clinically impairing diagnosis. Diagnostic formulations for this study were finalized during case presentation meetings led by experts in childhood anxiety, including one of the authors of the ADIS C/P. ADIS-C/P has demonstrated good psychometric properties, with excellent test–retest reliability (kappa = 0.65 to 0.88) and inter-rater agreement (kappa = 0.78 to 0.86) [67].

#### ***Child Behavior Checklist (CBCL***)[68]

Both parents completed the CBCL, a widely used and psychometrically sound parent-report measure of psychopathology in children and adolescents. CBCL consists of 112 items assessing externalizing and internalizing symptoms over the past 6 months and is rated on a three-point scale (i.e., 0 = "not true", 1 = "somewhat true", and 2 = "very true"). CBCL measure yields the following index scale categories: Total Problem scale, Internalizing scale, and Externalizing scale. For this study’s purposes, only parental reports on children’s internalizing and externalizing subscales were computed. The internalizing subscale allows parents to rate their child’s mood disturbance, including social withdrawal, somatic complaints, anxiety, and depression. The externalizing subscale assesses a child’s defiant behaviors and conduct problems, such as aggression, rule-breaking, temper tantrums, and delinquency. The psychometric properties of the CBCL are excellent and well established. Cronbach’s alpha internal consistency coefficients in previous research for the Internalizing scale and Externalizing scale scores ranged from 0.90 and 0.94. The convergent and divergent validity for the CBCL is also satisfactory [68, 69]. Internal consistency for the current sample for father-rated $(Cronbach \alpha = 0.84)$ and mother-rated internalizing scale $(Cronbach \alpha = 0.86)$ was good. Similarly, internal consistency for mother-rated $(Cronach \alpha = 0.88)$ and father-rated externalizing scale $(Cronbach \alpha = 0.90)$ was good.

#### ***Beck Anxiety Inventory (BAI)***[70]

The BAI is a self-report questionnaire designed to assess the presence of anxiety symptoms in adults. Both parents completed the BAI, querying the emotional (e.g., “terrified or afraid”), physiological (e.g., “difficulty in breathing”), and cognitive (e.g., “fear of dying”) aspects of anxiety they have experienced in the past month. Parents indicated the extent to which they were bothered by each of the 21-items in the past month on a 4-point Likert-type scale, ranging from 0 to 3 (0 = “Not at all”; 1 = “Mildly, but it did not bother me much”; 2 = “Moderately—it was not pleasant at times”; 3 = “Severely—it bothered me a lot”). The BAI is scored by summing each of the 21-items, yielding a total score that can range from 0 to 63. Good psychometric properties for the BAI have been reported in the previous research, showing high internal consistency, with Cronbach’s alpha ranging from 0.92 to 0.94, and good one-week test–retest reliability (0.75). The BAI has additionally demonstrated acceptable convergent and discriminant validity [71]. The current study’s internal consistency was excellent for mother-reported total BAI score $(\alpha = 0.94)$ and father-reported total BAI score $\left(\alpha =0.95\right).$

#### Family Accommodation Scale—Anxiety (FASA)[19]

FA was rated by both parents separately. FASA includes 13-items rated on a 5-point Likert-type scale from 0 to 4 (0 = “Never”; 1 = “One to three times a month”; 2 = “One to two times a week”; 3 = “Three to six times a week”; 4 = “Daily”). The first 9-items on the FASA assess the frequency of FA and yield the Total Accommodation score (9 items; range from 0 to 36), and subscale scores for Participation (items 1–5; range from 0 to 20) and Modification (items 6–9; range from 0 to 16). One item on the FASA (item 10) assesses parental distress associated with the accommodation (e.g., “Does helping your child in these ways cause you distress?”), whereas the last three items (items 11–13) assess negative short-term child reactions (e.g., anger) to parents not accommodating (e.g., “Has your child become angry/abusive when you have not provided assistance?”). The FASA has psychometrically good properties, showing excellent internal consistency, good test–retest reliability $(r = 0.79)$, as well as convergent and divergent validity, with Cronbach’s alphas ranging between 0.90 and 0.91 [19, 72]. In the current study, internal consistency for the father-reported Total Accommodation (9-items) score $(\alpha = 0.86)$ and mother-reported Total Accommodation score ($\alpha = 0.84)$ was good.

### Data Preparation

IBM Statistical Package of Social Sciences (SPSS, v. 26) was used to conduct all data preparation and statistical analyses. Before performing statistical analyses, all study variables were assessed for accuracy of data entry, the amount of missing data, and violations of the relevant statistical assumptions. Descriptive statistics assessed the frequency of all categorical variables, and means, standard deviations (SDs), and range were used to examine continuous variables. Further, patterns of missing values were examined, showing the missing data to be small and missing completely at random, which is why all subsequent analyses were conducted using all the available cases for each study measure as follows: for the CBCL Internalizing (*N* Fathers = 67; *N* Mothers = 68), for the CBCL Externalizing (*N* Fathers = 65; *N* Mothers = 67), and the BAI Total (*N* Fathers = 49; *N* Mothers = 67). Additionally, in order to examine if study variables met the statistical assumption of the normal distribution, skewness and kurtosis were calculated, with values of 0 indicating normally distributed data. Similarly, the presence of outliers was assessed for each study variable, with standardized values smaller than − 3.29 or greater than 3.29 as the criterion [73]. The assumption checks showed all variables to meet the assumptions, except for the following study variables: father and mother BAI Total scores, father and mother CBCL Internalizing scores, and father and mother CBCL Externalizing scores. In all of these variables, multiple outliers were detected. Consequently, in order to reach normality and remove the outliers, father-reported and mother-reported BAI Total score, CBCL Internalizing, and CBCL Externalizing variables were transformed using logarithmic transformation. The transformed variables removed the outliers and did not continue to violate the assumptions of normality after the transformation. Consequently, father-reported and mother-reported BAI Total, CBCL Externalizing, and CBCL Internalizing scores were used as transformed variables in all further analyses.

### Data Analytic Plan

Descriptive statistics, including mean and standard deviations, were used to describe fathers’ and mothers’ responses for each of the 13-items on the FASA scale. In addition to descriptive statistics, frequency counts were assessed to provide the number and corresponding percentage of fathers and mothers endorsing each of the 13-items on the 4-point Likert-type FASA scale. A paired-samples t-test was performed to assess for differences between fathers’ and mothers’ reports on the FASA, whereas Pearson’s bivariate correlations were calculated to assess the degree to which fathers’ reports on the FASA and mothers’ reports on the FASA are correlated. Next, independent samples t-test and one-way ANOVA were used to assess the demographic correlates of father-reported and mother-reported FA. Independent samples t-test was used to compare the mean scores of male and female children on father and mother reported FA, whereas a series of one-way ANOVAs were used to compare the means of the fathers’ and mothers’ education, racial, ethnic, employment, and income groups on FA Total and subscale variables. Pearson’s bivariate correlations were conducted to assess the associations between the correlates (CBCL Internalizing, CBCL Externalizing, BAI Total) of fathers’ and mothers’ reports on the FASA Total Accommodation score, Participation, Modification, Distress, and Consequences subscales. Similarly, Pearson’s bivariate correlation was also used to examine the relationship between fathers’ and mothers’ FA and child age.

## Results

### Frequency of Fathers’ FA and Mothers’ FA

Table 1 summarizes the means, standard deviations, and frequencies of fathers’ FA ratings and mothers’ FA ratings. Overall, almost all fathers reported engaging in FA, with $96.8 \%$ of fathers reporting participation in anxiety symptoms and $75.1\%$ of fathers reporting modifications to routines and schedules due to child anxiety symptoms. The most common form of participation in child symptoms by fathers was providing reassurance. Two-thirds of fathers $(n = 48; 69.5\%)$ reported providing reassurance three to six times a week $(36.2\%),$ and almost one-third reported doing so daily $(33.3\%)$. Modifying their everyday routines and doing things that are a child’s responsibility three to six times a week and on a daily basis were endorsed by 24.6% of fathers, respectively. Most fathers $(63.8\%)$ reported experiencing distress related to the need to accommodate, with $31.9\%$ of fathers reporting moderate to severe distress. Similarly, most fathers $(75.2\%)$ reported negative child consequences related to not being accommodated, with the most common being moderate to severe child distress when fathers did not assist their child $(n = 30; 43.4\%)$.

**Table 1:** Mean levels and frequency for each item and subscales of the father and mother reported FASA

Father						
Items		Never	1–3 times a month	1–2 times a week	3–6 times a week	Daily
	Mean (SD)	N (Percentage %)				
FASA Participation						
1. Providing reassurance	2.90 (1.02)	2 (2.9)	4 (5.8)	15 (21.7)	25 (36.2)	23 (33.3)
2. Providing items	1.57 (1.26)	18 (26.1)	15 (21.7)	18 (26.1)	13 (18.8)	5 (7.2)
3. Participating in behaviors	1.74 (1.39)	15 (21.7)	18 (25.4)	16 (23.1)	9 (13.0)	11 (15.9)
4. Assisting child in avoiding things	1.43 (1.03)	15 (21.7)	21 (30.4)	22 (31.9)	10 (14.5)	1 (1.4)
5. Avoiding things, places or people	.75 ( 0.99)	36 (52.1)	21 (30.4)	6 (8.7)	5 (7.2)	1 (1.4)
FASA Modification						
6. Modifying family routines	1.33 (1.45)	27 (39.1)	19 (27.5)	6 (8.7)	7 (10.1)	10 (14.5)
7. Doing things instead of child	1.16 (1.29)	30 (43.5)	14 (20.3)	8 (11.6)	12 (17.4)	5 (7.2)
8. Modifying work schedule	0.67 (1.08)	43 (62.3)	16 (23.2)	2 (2.9)	6 (8.7)	2 (2.9)
9. Modifying leisure activities	0.87 (1.11)	34 (49.3)	21 (30.4)	5 (7.2)	7 (10.1)	2 (2.9)

Items		No	Mild	Moderate	Severe	Extreme
FASA Distress						
10. Parent distress	1.01 ( .93)	25 (36.2)	22 (31.9)	18 (26.1)	4 (5.8)	–
FASA Consequences						
11. Child distress when FA not provided	1.39 (1.15)	20 (29.0)	17 (24.6)	19 (27.5)	11 (15.9)	2 (2.9)
12. Child angry or abusive	0.88 (1.20)	39 (56.5)	11 (15.9)	10 (14.5)	6 (8.7)	3 (4.3)
13. Child’s anxiety worsens	1.32 (1.22)	23 (33.3)	16 (23.2)	17 (24.6)	9 (13.0)	4 (5.8)

Mother						
Items		Never	1–3 times a month	1–2 times a week	3–6 times a week	Daily
	Mean (SD)	N (Percentage %)				
FASA Participation						
1. Providing reassurance	3.30 (.89)	–	3 (4.3)	11 (15.9)	17 (24.6)	38 (55.1)
2. Providing items	1.84 (1.57)	22 (31.9)	11 (15.9)	6 (8.7)	16 (23.2)	14 (20.3)
3. Participating in behaviors	2.45 (1.33)	7 (10.1)	11 (15.9)	15 (21.7)	16 (23.2)	20 (29.0)
4. Assisting child in avoiding things	2.30 (1.41)	9 (13.0)	14 (20.3)	13 (18.8)	13 (18.8)	20 (29.0)
5. Avoiding things, places or people	1.14 (1.28)	28 (40.6)	20 (29.0)	11 (15.9)	3 (4.3)	7 (10.1)
FASA Modification						
6. Modifying family routines	1.83 (1.54)	19 (27.5)	15 (21.7)	10 (14.5)	9 (13.0)	16 (23.2)
7. Doing things instead of child	1.64 (1.54)	24 (34.8)	13 (18.8)	9 (13.0)	10 (14.5)	13 (18.8)
8. Modifying work schedule	1.23 (1.48)	32 (46.4)	16 (23.2)	4 (5.8)	7 (10.1)	10 (14.5)
9. Modifying leisure activities	1.38 (1.36)	23 (33.3)	21 (30.4)	9 (13.0)	8 (11.6)	8 (11.6)

Items		No	Mild	Moderate	Severe	Extreme
FASA Distress						
10. Parent distress	1.68 (.93)	5 (7.2)	26 (37.7)	27 (39.1)	8 (11.6)	3 (4.3)
FASA Consequences						
11. Child distress when FA not provided	2.03 (1.08)	8 (11.6)	10 (14.5)	28 (40.6)	18 (26.1)	5 (7.2)
12. Child angry or abusive	0.94 (1.17)	36 (52.2)	12 (17.4)	12 (17.4)	7 (10.1)	2 (2.9)
13. Child’s anxiety worsens	1.81 (1.25)	15 (21.7)	12 (17.4)	17 (24.6)	21 (30.4)	4 (5.8)

*FASA* Family Accommodation Scale Anxiety [19]. Total Accommodation (Items 1–9), Participation (Items 1–5), Modification (Items 6–9), Distress (Item 10), Consequences (Items 11–13);

N_fathers_ = 69; N_mothers_ = 69

In this sample, all mothers reported at least some type of FA, with 99.4% of mothers reporting participation in symptom-related behaviors and 81% of mothers reporting modifications of family functioning, daily routines, or work schedules. The most common form of mothers’ participation was providing daily reassurance $(n=38, 55.1\%)$, as well as daily participation in behaviors related to child’s anxiety $(n=20, 29\%)$ and daily avoidance of doing things, meeting people, or going places because of child’s anxiety $(n=14, 20.3\%)$. Daily modification of family routines $(n=16, 23.2\%)$ and doing things that are child’s responsibility on a daily basis $(n=13, 18.8\%)$ were the most frequent forms of modification reported by mothers. Similarly, a total of 92.7% of mothers reported experiencing some levels of distress, with the majority of mothers reporting moderate to severe distress $(n=35, 66.7\%)$. Finally, 92.5% of mothers reported negative child consequences associated with not being accommodated, with most mothers reporting moderate to severe degree of child distress when accommodation was not provided $\left(n=46, 65.7\%\right).$ Moderate to a severe worsening of child’s anxiety $(n=38, 55\%)$ and child’s angry or abusive behavior $(n=19, 27.5\%)$ were also frequently reported negative consequences experienced by mothers who did not provide accommodation to their child’s anxiety symptoms.

### Comparison of Fathers’ and Mothers’ Reports on FA

Descriptive statistics, including mean, standard deviation, range, and a summary of the correlations and differences between fathers’ and mothers’ reports on the FASA are presented in Table 2.

**Table 2:** Correlations and differences between fathers’ and mothers’ FASA reports

	Father		Mother			
Variable	Mean (SD)	Range	Mean (SD)	Range	*Pearson r*	* t*
FASA Total	12.26 (7.69)	0–31	17.11 (8.39)	3–36	0.442**	− 4.73**
Participation	8.26 (4.42)	0–18	11.04 (4.89)	2–20	0.391**	− 4.48**
Modification	4.00 (3.85)	0–13	6.07 (4.59)	0–16	0.406**	− 3.70**
Distress	1.01 (0.93)	0–3	1.68 (0.93)	0–4	0.277*	− 4.94**
Consequences	3.57 (3.16)	0–12	4.78 (2.65)	0–12	0.521**	− 3.46**

Bold values indicate statistically significant findings

Range of possible scores on FASA: Total Accommodation: 0–36, Participation 0–20, Modification: 0–16, Distress: 0–4, Consequences: 0–12.

*N*_*father*_ = *69; N*_*mother*_ = *69*

*FASA* Family Accommodation Scale—Anxiety [19]

*p < 0.05; **p < 0.01

Pearson’s correlation coefficients indicated significant positive correlations between fathers’ and mothers’ reports on the FASA Total Accommodation, as well as its subscales. The degree of correlation between fathers’ and mothers’ FASA reports was overall moderate, whereas a low degree of correlation was found between fathers’ and mothers’ reports on the FASA Distress subscale. Assessment of differences in means between fathers’ and mothers’ FA revealed that overall, mothers’ FASA Total Accommodation scores were significantly higher than were fathers’ scores on FASA Total Accommodation. Similarly, mothers’ scores on the FASA subscales, including Participation, Modification, Distress, and Consequences subscales, were significantly higher than fathers’ scores (see Table 2).

### Demographic Correlates of Fathers’ and Mothers’ Accommodation

A series of one-way ANOVAs were conducted to examine the sociodemographic correlates, including fathers’ and mothers’ education level, race, ethnicity, employment status, and annual household income, in relation to fathers’ and mothers’ reports on the FASA Total Accommodation, as well as Participation, Modification, Distress, and Consequences subscales. No statistically significant links emerged between any demographic correlates and fathers’ and mothers’ reports on the FASA Total Accommodation, Participation, Modification, Distress, and Consequences (see Table 3). In addition, independent samples t-tests were conducted to examine whether fathers’ and mothers’ accommodation differed based on child sex. Only one significant difference emerged: mothers of male children reported higher distress related to accommodation $\left(M=1.85, SD=0.95\right)$ than did mothers of female children $(M=1.40, SD=00.84)$, $t(67)=2.05, p = 0.04$ (see Table 3). Apart from this, results showed no significant differences in fathers’ or mothers’ FASA (Total or subscale) scores across child sex.

**Table 3:** Demographic correlates of father and mother reported accommodation

Variable	Child sex	Education groups	Race	Ethnic groups	Employment status	Income
	$t$	$F-\mathrm{Values}$				
Father FASA Total	1.09	0.673	0.421	0.046	2.60	0.330
Father Participation	1.00	1.00	0.598	0.681	2.99	0.444
Father Modification	1.02	0.570	0.613	0.265	1.51	0.377
Father Distress	1.68	1.15	0.979	0.002	1.69	0.291
Father Consequences	1.82	0.710	0.656	0.018	0.002	0.609
Mother FASA Total	0.179	1.48	0.050	0.531	2.10	1.40
Mother Participation	− 0.744	1.77	0.260	0.917	0.231	2.17
Mother Modification	1.12	0.749	0.024	0.097	2.20	1.42
Mother Distress	2.05*	1.67	1.18	0.867	0.079	1.80
Mother Consequences	− 0.080	1.68	1.28	0.624	1.04	1.22

Bold value indicates a statistically significant finding

*FASA* Family Accommodation Scale—Anxiety [19]

Education consisted of the following groups: 1st group: high school; 2nd group: some college; 3rd group: associate’s; 4th group: bachelor’s; 5th group: master’s; 6th group: Ph.D.; 7th group: Advanced

Racial groups consisted of the following: 1st group: White; 2nd group: Black or African American; 3rd group: Asian; 4th group: American Indian/Alaska Native; 5th group: Multiracial

Ethnic groups: 1st group: non-Hispanic or Latino; 2nd group: Hispanic or Latino

Employment groups: 1st group: Employed; 2nd group: Unemployed

Income consisted of the following groups: 1st group: $21,000–$40,999; 2nd group: $41,000–$60,999; 3rd group: $61,000–$80,999; 4th group: $81,000–$99,000; 5th group: $100,000–$124,999; 6th group: $125,000–$149,000; 7th group: $150,000 + . *N*_*father*_ = *69; N*_*mother*_ = *69*

*p < 0.05; **p < 0.01

### Psychosocial Correlates of Fathers’ and Mothers’ Accommodation

Pearson correlation coefficients between fathers’ FASA ratings (Total and subscales) and clinical characteristics are summarized in Table 4. Overall, results indicated a significant positive correlation between father reported FASA Total Accommodation scores and fathers’ ratings on child’s internalizing symptoms (Father CBCL Internalizing) and child’s externalizing symptoms (Father CBCL Externalizing). A positive, significant correlation emerged between fathers’ FASA Total Accommodation scores and fathers’ self-reported anxiety symptoms (Father BAI Total), whereas a non-significant correlation was found between fathers’ FASA Total Accommodation scores and child age.

**Table 4:** Pearson’s bivariate correlations between father-reported measures

Variable	1	2	3	4	5	6	7	8	9
1. Father FASA Total									
2. Father Participation	0.939**								
3. Father Modification	0.919**	0.727**							
4. Father Distress	0.624**	0.610**	0.545**						
5. Father Consequences	0.717**	0.661**	0.673**	0.601**					
6. Father Internalizing	0.457**	0.387**	0.469**	0.087	0.363**				
7. Father Externalizing	0.403**	0.324**	0.431**	0.211	0.436**	0.559**			
8. Father BAI Total	0.314*	0.213	0.373**	0.174	0.358**	0.458**	0.424**		
9. Child’s age	− 0.159	− 0.184	− 0.107	− 0.208	− 0.280*	0.007	− 0.169	− 0.121	

Bold values indicate statistically significant correlations

*FASA* Family Accommodation Scale—Anxiety [19], including Participation, Modification, Distress, and Consequences, *CBCL* Child Behavior Checklist [68]; BAI: Beck’s Anxiety Inventory [70]

*p < 0.05; **p < 0.01

A summary of mothers’ FASA ratings, including Total Accommodation and subscales, is presented in Table 5. Mothers’ reports on FASA Total Accommodation scale were significantly and positively associated with mothers’ reports on child’s externalizing (Mother CBCL Externalizing) but not child’s internalizing symptoms (Mother CBCL Internalizing). A non-significant correlation emerged between mothers’ reports on FASA Total Accommodation and mothers’ self-reported anxiety symptoms (Mother BAI Total), whereas a significant, negative correlation was found between child age and mothers’ reports on FASA Total Accommodation.

**Table 5:** Pearson’s bivariate correlations between mother-reported measures

Variable	1	2	3	4	5	6	7	8	9
1. Mother FASA Total									
2. Mother Participation	0.892**								
3. Mother Modification	0.876**	0.563**							
4. Mother Distress	0.490**	0.464**	0.401**						
5. Mother Consequences	0.641**	0.543**	0.591**	0.489**					
6. Mother Internalizing	0.215	0.116	0.268 *	0.051	0.145				
7. Mother Externalizing	0.296*	0.148	0.384 **	0.105	0 .453**	0.385**			
8. Mother BAI Total	0.156	0.158	0.117	0.099	0.194	0.151	0.350**		
9. Child’s age	− 0.327**	− 0.303*	− 0.274**	− 0.004	− 0.337**	0.013	− .0.190	0.080	

Bold values indicate statistically significant correlations

*FASA* Family Accommodation Scale—Anxiety [19], including Participation, Modification, Distress, and Consequences, *CBCL* Child Behavior Checklist [68]; BAI: Beck’s Anxiety Inventory [70]

*p < 0.05; **p < 0.01

## Discussion

### Frequency of Fathers’ FA and Mothers’ FA

The current study is the first to assess the frequency of fathers’ FA using FASA measure, and the first to primarily focus on fathers by examining correlates of fathers’ participation in behaviors related to child anxiety, fathers’ modification of family routines and schedules, fathers’ distress related to the need to accommodate, and fathers’ reports of child distress and anger when accommodation is not provided. Overall, results indicate that FA is highly prevalent among both fathers and mothers of clinically anxious children and adolescents, with almost all fathers and mothers participating in symptom-related behaviors and the majority of both fathers and mothers modifying family routines and schedules due to child’s anxiety. A higher percentage of mothers than fathers reported distress relating to the need to accommodate. Likewise, more mothers than fathers reported short-term negative child consequences when not providing accommodation. In line with our hypothesis and consistent with previous research [19, 21, 23, 26], these findings confirm high accommodation rates in mothers and suggest similarly high, albeit slightly lower, accommodation rates in fathers of children with anxiety disorders. Additionally, findings indicate that the most frequently endorsed forms of accommodation among fathers and mothers were the provision of reassurance and participation in behaviors related to child anxiety. These findings are also in line with previous studies indicating that provision of reassurance is the most common form of FA [31, 36, 40].

### Comparison Between Fathers’ FA and Mothers’ FA

Despite these similarities, fathers reported somewhat lower rates of accommodation than did mothers. This pattern held across overall accommodation as well as the subscales for participation, modification, distress, and consequences. This pattern of findings confirms our hypothesis and is consistent with previous research showing discrepancies among informants’ reports in the assessment of child and adolescent anxiety symptoms [60, 63], with mothers consistently reporting slightly higher clinical symptoms in their children than fathers [59–61]. However, it is important to bear in mind that in the current study, fathers and mothers were each reporting on *their own* accommodation. As such, differences between father and mother ratings can reflect actual real-life differences in the frequency of engaging in accommodation as well as differences in perceptions or response styles.

The results further indicate an overall moderate degree of correlation between fathers’ and mothers’ reports on the FASA. This pattern held across the FASA subscales, showing a significant, moderate degree of correlation between fathers’ and mothers’ reports on the subscales for participation in symptom-related behaviors, modification of family routines, and negative consequences related to accommodation. These findings are consistent with earlier research showing moderate associations between fathers’ and mothers’ reports on the scope and interference of accommodation in pediatric anxiety disorders [21] and with the moderate correlations between parents found in studies assessing multi-informant perspectives on clinically anxious youth [61, 62]. However, at odds with our hypothesis is the finding of a low degree of correlation between mothers and fathers on levels of distress relating to the accommodation. Mothers reported greater distress than did fathers. This is, however, in line with previous research indicating discrepancies in multi-informants’ perceptions of the extent to which child anxiety symptoms are distressing [60]. Greater distress related to accommodation in mothers than fathers may be related to a higher degree of sensitivity in mothers to children’s distress [41, 58], to mothers holding negative beliefs about children’s experience of anxiety symptoms [42], and to the possibility that mothers may accommodate their anxious children as a result of own distress [23, 58, 62]. It is also plausible that providing accommodation is less distressing to fathers than it is to mothers, and this possibility calls for additional research into the motivations and burden associated with FA.

The integration of findings showing both moderate degrees of correlation and small but significant differences between fathers’ and mothers’ reports of various aspects of FA suggests that both parents become entangled in frequent accommodation but do so to somewhat differing degrees and have different perspectives on the ramifications of not accommodating [62]. The fact that fathers’ and mothers’ reports were not more strongly correlated could also be due to the situation specificity [64] or to bias in reporting linked to parents’ wish to present themselves favorably [40]. Indeed, in research comparing fathers’ and mothers’ reports of child anxiety, fathers have generally rated their children as less anxious than mothers [59]. The particularly low degree of correlation on reports of distress related to the accommodation highlights the importance of how parents’ own distress may influence their reports of child anxiety symptoms and FA and should be taken into consideration in future assessments.

### Correlates of Fathers’ and Mothers’ FA

Given that participants in our study were clinically anxious children living with both of their parents and that each parent reported on their own accommodation of their child's anxiety symptoms, we investigated demographic and clinical correlates of both fathers' and mothers' FA. Our results on child-related clinical correlates indicated that fathers' overall accommodation was positively correlated with their reports on children's internalizing and externalizing symptoms, whereas mothers' overall accommodation was significantly associated with children's externalizing symptoms but not with internalizing symptoms. However, despite the non-significant association with mothers' overall accommodation, we found a positive, significant correlation between mothers' reports on the FASA modification subscale and mothers' reports of child internalizing symptoms. These findings are consistent with our hypotheses and with previous research [25, 31, 36–39, 41], suggesting that both fathers and mothers in our sample may be more prone to accommodate in response to their child's externalizing symptoms (e.g., rage attacks, outbursts, and temper tantrums). However, whereas fathers who perceive their child's internalizing symptoms may accommodate more in general, our findings suggest that mothers who view their child as anxious, depressed, or socially withdrawn are more likely to accommodate specifically by modifying family routines, work schedules, and leisure activities.

When examining parent-related clinical correlates, our results on fathers indicated that fathers who rated themselves as more anxious were also more likely to engage in higher levels of accommodation, including modification of family routines and to report negative child consequences, such as temper tantrums, when they did not accommodate. Although our findings on fathers are in line with our hypothesis, they are also at odds with the findings of a recent study that did not find a significant association between fathers’ anxiety symptoms and fathers’ overall accommodation in a smaller sample of 41 fathers [41]. In contrast to fathers, our results on mothers indicated that mothers’ overall accommodation, including participation, modification, distress, and consequences, was not related to mothers’ self-reported anxiety symptoms. These findings were unexpected and are in contrast with previous research linking maternal anxiety to higher FA [34, 42, 43]. Considering that child distress has been identified as a major motivating factor driving accommodation, it may be that mothers in our sample were more motivated to accommodate in response to their child’s distress instead of their own distress or their own difficulties in emotional regulation [42, 43]. In fact, a recent study exploring maternal distress and emotional regulation related to FA found that mothers’ perception of child distress related to anxiety was more influential than mothers’ own anxiety and depressive symptoms in predicting mothers’ FA [41].

When examining the link between fathers’ and mothers’ FA and child age, our results showed a non-significant association with fathers’ overall accommodation, but a significant, negative association with mothers’ overall accommodation, participation in child’s symptom-driven behaviors, and modification of family routines. Results also indicated that child age was significantly and negatively linked to both fathers’ and mothers’ reports on the negative consequences of not accommodating, suggesting that both parents may be more likely to accommodate younger children when they become distressed or angry when parents do not accommodate. Despite the non-significant association with fathers’ overall accommodation, our findings are consistent with previous research on mothers [21, 25, 36, 41], suggesting that younger children rely more heavily on their parents for help and that parents, in turn, perceive younger children as more in need of their accommodation. Previous studies have also indicated that younger children rely more heavily on their parents for help in anxiety-provoking situations and engage in externalizing behaviors, such as rage attacks, outbursts, and temper tantrums to elicit accommodation from parents [24, 33, 39].

Our results on demographic correlates revealed no effect of child sex on fathers’ or mothers’ reports of accommodation, suggesting that parents similarly accommodate female and male children. These findings are in line with our hypotheses and are consistent with previous studies showing no significant link between FA and child sex [21, 25, 36, 41]. However, contrary to our expectation, mothers’ reports on the distress subscale differed based on child sex, suggesting that mothers of male children experienced significantly greater distress related to accommodation than mothers of female children. Considering past mixed findings on FA and child sex [19, 21, 25, 36], more research is needed before definite conclusions are drawn on the effect of child sex on FA. In addition, no significant relations were found between fathers’ and mothers’ reports on the various aspects of accommodation and fathers’ and mothers’ demographic variables, including education, race, ethnicity, employment status, and annual household income. Considering that this is the first study to explore fathers’ demographic correlates in relation to FA, replicating these findings is necessary before conclusions are drawn. Additionally, considering that our sample was relatively homogeneous, research with more diverse samples is required before the findings can be generalized.

### Clinical Implications

The present study is the first to specifically focus on rates of fathers’ accommodation of children with anxiety disorders, as well as the first to assess correlates of fathers’ ratings on the FASA subscales, including child internalizing and externalizing symptoms, fathers’ own anxiety symptoms, and child and parent-related demographic variables. Results indicate that accommodation is common in fathers of children with anxiety disorders. Considering that the assessment of FA has thus far primarily relied on mothers’ reports, the assessment of FA may be significantly enhanced through the collection of data from multiple informants, especially those of fathers and children. Integrating fathers’, mothers’, and children’s perspectives might have greater treatment implications as it allows clinicians to create a more accurate clinical picture.

The integration of data collected from multiple informants for the assessment of FA may significantly improve parent-based interventions designed to address the accommodation. One such intervention is SPACE (Supportive Parenting for Anxious Childhood Emotions) that focuses on helping parents to identify and reduce FA and to increase supportive responses that acknowledge the child’s distress while conveying confidence in the child’s ability to cope with and tolerate anxiety [74]. In a recent randomized controlled trial, SPACE was found to be as efficacious as cognitive-behavioral therapy for the treatment of pediatric anxiety disorders [49]. Treatments such as SPACE that focus on FA may benefit from improvements in the assessment of FA through multi-informant methodologies. Examining fathers’ reports would allow parents undergoing treatment to discuss differences in perceptions of FA and would aid parents and clinicians in becoming more aware of different ways family members respond to the anxiety symptoms.

## Limitations and Future Directions

While the present study adds to the growing body of literature on FA in pediatric anxiety disorders, the results should be interpreted carefully as several limitations of this research study warrant attention. First, the cross-sectional nature of the present study precludes drawing any causal inferences, and future studies would benefit from longitudinal designs that can provide more information about parental dynamics emerging within families of clinically anxious children in general and fathers’ involvement in particular. Second, although the sample size in the present study was adequate and consistent with sample sizes reported in previous FA studies [21, 37, 40], future studies should collect data from larger samples that might allow for more advanced statistical analyses of the relationship between the relevant variables and their contribution to fathers’ accommodating behaviors. Third, the results need to be considered in light of the demographic limitations, as the sample was relatively homogenous. Specifically, the sample was predominantly non-Hispanic, White, and of fairly high socioeconomic status. As this sample consisted only of children living in two-parent, heterosexual families, future studies should include more heterogeneous samples to determine whether the results are generalizable to a more diverse population. Considering that the sample in this study did not include children living in single-parent households, investigating the parental accommodation of children living in single-parent households should be considered for future research. Finally, future research should further explore the ways in which parental characteristics, such as parents’ own symptomatology, may influence and bias parental ratings of FA. In a recent study, Zilcha-Mano et al. [75] explored the relationship between mothers’ and children’s agreement and disagreement on FA and subsequent treatment outcomes, showing that parent–child agreement and disagreement on FA are significant predictors of subsequent reduction in the severity of anxiety symptoms. Future studies could also investigate correlations and differences in FA between fathers and mothers and between fathers and children, as this may have further implications for subsequent clinical outcomes for children with anxiety disorders.

## Summary

The present study primarily assessed fathers’ FA reports using the FASA measure. Our study specifically investigated the frequency and correlates of fathers’ FA by examining fathers’ participation in symptom-driven behaviors, modification of family routines, distress related to accommodation, and negative consequences when not providing accommodation to their child. Our study additionally compared fathers’ and mothers’ reports of FA and included frequency and correlates of mothers’ accommodation. Fathers reported highly prevalent accommodation, and their reports were significant and positively correlated with child internalizing symptoms, externalizing symptoms, and fathers’ own anxiety symptoms. Similarly, mothers reported highly prevalent FA, and their reports were positively associated with child externalizing and internalizing symptoms, but not mothers’ own anxiety symptoms. Fathers endorsed a slightly lower level of accommodation than mothers, but a moderate degree of correlation was found between fathers’ and mothers’ reports on accommodation of clinically anxious children. Obtaining reports from both fathers and mothers may enhance the assessment of accommodation in families of clinically anxious children in both clinical and research settings.
